# *Mycoplasma ovipneumoniae* induces caspase-8-dependent extrinsic apoptosis and p53- and ROS-dependent intrinsic apoptosis in murine alveolar macrophages

**DOI:** 10.1080/21505594.2021.1984714

**Published:** 2021-10-22

**Authors:** Jing Chen, Yi Zhou, Erpeng Zhu, Peng Yang, Mei Li, Shuangxiang Zhang, Jun Yue, Ming Wen, Kaigong Wang, Zhentao Cheng

**Affiliations:** a Department of Veterinary Medicine, College of Animal Science, Guizhou University, Guiyang, China; b Key Laboratory of Animal Diseases and Veterinary Public Health of Guizhou Province, College of Animal Science, Guizhou University, Guiyang, China; c The Laboratory of Veterinary Medicine, Animal Disease Prevention and Control Center of Guizhou Province, Guiyang, China

**Keywords:** *Mycoplasma ovipneumoniae*, apoptosis, caspase-8, p53, ROS, proinflammatory cytokine

## Abstract

*Mycoplasma ovipneumoniae (MO)* is a principle causative agent of chronic respiratory disease in ruminants, including sheep, goats, and deer, posing a great threat to the ruminant industry worldwide. However, the pathogenesis of *MO* infection still remains not well understood and needs further clarification. Here we report a time-dependent apoptosis in cultured murine alveolar macrophage (MH-S) cell lines in response to *MO* infection *in vitro*. Mechanistically, *MO* infection activated apoptosis in MH-S cells through caspase-8-dependent extrinsic pathway and through tumor protein 53 (p53)- and reactive oxygen species (ROS)-dependent intrinsic mitochondrial pathways. Moreover, *MO* infection promoted both transcription and translation of proinflammatory cytokine genes including *interleukin-1β* (*IL-1β), IL-18*, and *tumor necrosis factor-α* (*TNF-α*), in a caspase-8-, p53-, and ROS-dependent manner, implying a potential link between *MO*-induced inflammation and apoptotic cell death. Collectively, our results suggest that *MO* infection induces the activation of extrinsic and intrinsic apoptotic pathways in cultured MH-S cells, which is related to upregulated expression of proinflammatory cytokines. Our findings will contribute to the elucidation of pathogenesis in *MO* infection and provide valuable reference for the development of new strategies for controlling *MO* infection.

## Introduction

*Mycoplasma ovipneumoniae* (*MO*), belonging to the genus *Mycoplasma* within the family *Mycoplasmataceae*, is a wall-less microorganism with minute genomes consisting of circular double-stranded DNA and is generally recognized as the primary causative agent of chronic non-progressive pneumonia in sheep and goats [1,2]. However, *MO* has a wide host range from the subfamily *Caprinae* species to non-*Caprinae* species including beira antelope, cattle, mule, and several deer species [3–6]. *MO*-induced pneumonia is prevalent in many countries with a high mortality rate, thus posing a great threat to industry of ruminants worldwide [7,8]. *MO* was first identified in 1963 [9] and has been studied for decades [2,6,10]. However, the molecular pathogenesis of *MO* is still not well understood, probably owing to the fact that its hypercritical nature renders it difficult to be cultured, prior to the rapid development of molecular biology techniques [11,12].

Apoptosis is an active cell death process regulated by a series of genes to maintain cellular homeostasis and plays crucial roles in both physiological and pathological processes, including embryonic development and morphogenesis, immune defenses, and pathogens-induced cell damage [13–17]. The mechanisms of apoptosis are complex, among which two classical pathways are mitochondrial events-mediated intrinsic pathways and cellular transmembrane receptors, such as tumor necrosis factor receptor (TNFR) superfamily-mediated extrinsic pathways [18]. Besides, endoplasmic reticulum stress (ERS) and ERS-driven unfolded protein response (UPR) pathways also play important roles in induction of intrinsic apoptosis [19,20]. The transmembrane receptors-, mitochondria-, and ERS-mediated apoptosis are dependent on the initial activation of caspase-8, caspase-9, and caspase-12, respectively, followed by activation of the corresponding caspase cascades conveying the apoptotic signals sequentially, thus resulting in cell death via degradation of certain cellular targets [18,20,21]. Increasing evidence reveals that apoptosis has been implicated in the infection and pathogenicity of a high number of pathogens, such as severe acute respiratory syndrome coronavirus-2 (SARS-CoV-2), highly pathogenic avian influenza virus (HPAIV), and *Shigella flexneri* [16,22,23]. *MO* infection also induces reactive oxygen species (ROS)-dependent and mitogen-activated protein kinases (MAPK)-caspase-3-mediated mitochondrial apoptotic pathways in sheep bronchial epithelial cells [24,25]. Further study suggests that the capsular polysaccharide of *MO* activates caspase-8-dependent extrinsic apoptosis and induces ROS-dependent intrinsic apoptosis via c-Jun N-terminal Kinase (JNK)/p38 MAPK pathways in sheep airway epithelial cells [26]. However, the concrete mechanisms of *MO*-induced apoptosis need further clarification.

As for multicellular organisms, apoptosis and inflammation are of great significance in maintaining cellular homeostasis and defending the body from invading foreign microorganisms, respectively. But actually, apoptosis and inflammation share many functional cross-talks during their progression [27–29]. It is known to all that apoptosis does not cause inflammation directly. However, many stimuli-induced proinflammatory cytokines, including tumor necrosis factor-α (TNF-α) and interleukin-1β (IL-1β) have been implicated in the induction of apoptosis [30–32], by which the inseparable relationship between apoptosis and inflammation has been evidenced. Although *MO* has been demonstrated to induce apoptosis and inflammation, the relationship between *MO*-mediated inflammation and apoptosis remains unclear.

In this study, based on the established murine alveolar macrophage (MH-S) cell model for *MO* infection *in vitro*, we found that *MO* infection induces apoptosis in cultured MH-S cells through caspase-8-dependent extrinsic pathway and through tumor protein 53 (p53)- and ROS-dependent intrinsic pathway. Furthermore, we suggest that extrinsic and intrinsic apoptosis caused by *MO* infection are associated with increased expression of proinflammatory cytokines, including IL-1β, IL18, and TNF-α. These findings shed new light on the pathogenesis of *MO* and meanwhile provide a scientific basis for the development of new strategies for controlling *MO* infection.

## Materials and methods

### Reagents and antibodies

RNAiso Plus reagent (9108) and TB Green *Premix Ex Taq*^TM^ II (RR820A) were purchased from TaKaRa. The TUNEL detection kit (11,684,817,910) was purchased from Roche. Annexin V: FITC apoptosis detection kit I (556,547) was purchased from BD. Cell counting kit-8 kit (C0037), ROS detection kit (S0033S), BeyoECL Star kit (P0018AS), and p53 inhibitor Pifithrin-α (Pft-α, S1816) were purchased from Beyotime. IL-1β (JYM0531Mo), IL-18 (JYM0543Mo), and TNF-α (JYM0218Mo) ELISA kits were purchased from JYM Biotech. ROS inhibitor N-acetyl-L-cysteine (NAC, HR8848) was obtained from Beijing Biolab. Caspase-8 inhibitor Z-IETD-FMK (HY-101297) was obtained from MedChemExpress.

Rabbit anti-p53 (acetyl K370) antibody (ab183544) was purchased from Abcam. Rabbit anti-caspase-12 antibody (NBP1-76801SS) was purchased from Novus Biologicals. Rabbit anti-caspase-8 (D35G2) antibody (4790 T), rabbit anti-Bax antibody (2772 T), rabbit anti-Bcl-2 antibody (3498 T), and rabbit anti-β-actin antibody (4970 T) were purchased from Cell Signaling Technology. Horseradish peroxidase (HRP)-labeled goat anti-rabbit IgG(H + L) antibody (A0208) was obtained from Beyotime.

### Propagation of MO

The type of strain of *MO* (Y98 strain) obtained from the China Institute of Veterinary Drug Control (Beijing, China) was propagated in sterile modified Hayflick’s medium (prepared with a 15:4:1 ratio of PPLO medium, premium horse serum, and auxiliary liquid) containing 0.002% phenol red at 37°C. After incubation for 5–7 days, the mycoplasma was passed. The titer of *MO* culture was measured with color-changing units (CCU) assays and expressed as CCU/mL as previously described [33,34]. The multiplicity of infection (MOI) was calculated according to the obtained *MO* titers and the number of cells when seeded.

### Cell culture, infection, and biochemical intervention

MH-S cells (ATCC, CRL-2019) were cultured in the Roswell Park Memorial Institute (RPMI) 1640 medium (Sigma, R8758), containing 10% (v/v) fetal bovine serum (FBS, Gbico, 10,091,148) and 1% (v/v) penicillin–streptomycin (Gibco, 15,140,122). Cultured cells were maintained in a HF240 incubator (Heal Force) at 37°C with 5% CO_2_ and passaged when they reach 80%–90% confluence. For infection, 10 MOI of *MO* was collected by centrifugation for 10 min at 12,000 r/min and resuspended with fresh media, and then the resultant mixture was inoculated into the MH-S cells and further maintained at 37°C with 5% CO_2_ for the indicated time.

To investigate the effect of p53, ROS, and caspase-8 inhibitors on expression of apoptosis-related factors, apoptotic rates, and proinflammatory cytokines in *MO*-infected MH-S cells, cells plated in 24-well plates were cultured for 4 h at 37°C with 5% CO_2_, and then, respectively, pretreated with serial concentrations of p53 inhibitor Pft-α (5 μM, 15 μM, 25 μM), ROS inhibitor NAC (1:400, 1:800, 1:1 600), and caspase-8 inhibitor Z-IETD-FMK (10 μM, 20 μM, 30 μM) for 30 min, followed by infection with 10 MOI of *MO*. The cells were further grown in a maintenance medium without the indicated chemicals for 24 h and then collected for the following assays.

### Transmission electron microscopy (TEM)

Cells collected at 24 hours post-infection (hpi) were fixed in 2.5% glutaraldehyde (Solarbio, P1126) for 30 min at room temperature (RT) and then scraped gently for centrifugation, followed by three washes with 0.1 M phosphate buffer (PB, pH7.4), 15 min each time. The cells were further fixed with 1% osmic acid in 0.1 M PB for 2 h at RT. After three washes with 0.1 M PB, the cells were dehydrated in a graded ethanol series (50%, 70%, 80%, 90%, 95%, and 100%) and 100% acetone in turn, 15 min each time. After gradient penetration with embedding agent (incubated in a 1:1 mixture of 812 embedding resins and acetone for 2–4 h, a 2:1 mixture of resin and acetone overnight, and absolute resin for 5–8 h in turn), samples were embedded at 60°C for 48 h and then sectioned (60–80 nm thick) with an ultra-thin microtome (Leica, EM UC7), followed by staining with 2% uranyl acetate saturated alcohol solution and lead citrate, each staining for 15 min. The sections were dried overnight at RT and observed under a Tecnai G2 F20 S-Twin transmission electron microscope (FEI). TEM were also used for ultrastructural observation of the *MO*-infected MH-S cells.

### Confocal fluorescence microscopy (CFM)

CFM was also performed to detect the *MO* infection in MH-S cells. *MO* was prestained with 1,1ʹ-dioctadecyl-3,3,3ʹ,3ʹ-tetramethylindocarbocyanine perchlorate (DilC18(3)/Dil, Solarbio, D8700), a widely used lipophilic membrane dye showing orange-red fluorescence. Briefly, the *MO* culture was mixed with Dil and incubated in a 37°C incubator for 15 min. After centrifugation at 5 000 r/min for 10 min, the *MO* pellets were rinsed with PBS for 5 times and then resuspended with culture media for infection of MH-S cells. At 24 hpi, cells were fixed with 4% paraformaldehyde for 10 min at RT. After three washes with PBS, the cells were further subjected to nuclear staining with DAPI (blue) for 5 min. The fluorescent signals were observed under a TCS SP8 confocal microscope (Leica).

### Terminal deoxynucleotidyl transferase-mediated dUTP-biotin nick end labeling (TUNEL) assays

MH-S cells were plated in 6-well microplates with cover glasses put in advance and cultured in a 5% CO_2_ incubator at 37°C for 2 h, and then subjected to *MO* infection (10 MOI) or mock infection. Cells at 6 hpi, 12 hpi, and 24 hpi were collected for TUNEL assays to detect apoptosis according to instructions for the TUNEL kit (Roche). Briefly, the cells were fixed with 4% paraformaldehyde for 30 min at RT and then penetrated with 0.2% (v/v) Triton X-100 for 5 min, followed by incubation in a mixture containing a 1:9 ratio of Reagent 1 (TdT) and Reagent 2 (dUTP) at 37°C for 60 min. After staining with DAPI, cover glasses were mounted on glass slides and then sealed for observation under a fluorescence microscope (Olympus, IX73). The fluorescence of cells in each group is analyzed with ImageJ software, and results are expressed as percent of TUNEL-positive cells.

### Flow cytometry

MH-S cells plated in 6-well microplates were cultured at 37°C with 5% CO_2_ for 2 h and then subjected to *MO* infection (10 MOI) or mock infection. Cells at 6 hpi, 12 hpi, and 24 hpi were collected for determination of apoptosis rates in *MO*-infected MH-S cells by flow cytometry. The effect of p53, ROS, and caspase-8 inhibitors on the apoptotic rates in the *MO*-infected MH-S cells at 24 hpi was also determined. Briefly, the cells cultured in the 6-well plates were carefully washed once with pre-cooled PBS and digested with 0.25% trypsin, and then transferred into a tube containing pre-collected culture media. After washing once in 3 mL of pre-cooled PBS, the pellets were resuspended with 300 μL binding buffer. About 5 μL Annexin V-FITC reagent was added to the resultant suspensions and incubated in the dark for 10 min, followed by the addition of 5 μL PI to the mixture for incubation in the dark for 5 min. The obtained solution was subject to flow cytometry analysis on a FACSCalibur flow cytometer (BD).

### PCR and qRT-PCR assays

PCR assays were performed as previously described [35] to detect the *MO* infection in MH-S cells. Briefly, the total DNA of the collected cells at 24 dpi was extracted according to instructions of the DNA extraction kit (Beijing Anheal laboratories, YDNA-TQ-20190713P), as template for amplifying specific *heat shock protein 70* (*Hsp70*) genes of *MO*. Then, the amplified products were identified on 1% agarose gels.

Mock- and *MO*-infected MH-S cells were, respectively, collected at 0 hpi, 6 hpi, 12 hpi, and 24 hpi and then subject to qRT-PCR assays as previously described [36]. Briefly, the total RNA of each sample was extracted in accordance with instructions for the RNAiso Plus kit (Takara), and then reversely transcribed into cDNA for relative quantification of the *p53, caspase-8*, and *caspase-12* mRNAs. Moreover, specific inhibitor- and solvent-pretreated *MO*-infected MH-S cells, as well as mock-infected cells collected at 24 hpi were subject to determination of transcriptional levels of apoptosis-related genes (*caspase-3, caspase-9, Bcl-2-associated X protein (Bax), B-cell lymphoma-2 (Bcl-2*), and *apoptosis inducing factor (AIF)*) and proinflammatory cytokine genes (*IL-1β, IL-18*, and *TNF-α*) by qRT-PCR assays. Each sample was set up in triplicate and assayed on a Bio-Rad real-time PCR apparatus (1,855,200). The relative mRNA expression levels of the indicated genes normalized to *β-actin* gene were calculated by the 2^−ΔΔCT^ method. The primer information is listed in Table 1.

**Table 1.** Primer information

Genes	Sequence (5ʹ ~ 3ʹ)	GenBank Nos.	Size (bp)
*Hsp70*	Forward: CCTGCTCCTCGTGGTCTTC Reverse: GGGCTGCTTGCTCAATTTGGT	HM047293.1	397
*p53*	Forward: CCGGCTCTGAGTATACCACCATCC Reverse: TGGTAAGGATAGGTCGGCGGTTC	M13874.1	87
*caspase-8*	Forward: CACAAGAAGCAGGAGACCATCGAG Reverse: GCAGTCTAGGAAGTTGACCAGCAG	NM_001277926.1	150
*caspase-12*	Forward: ACAAAGGCCCATGTGGAGAC Reverse: AAGAGGGAACCAGTCTTGCCT	AY675224.1	92
*caspase-3*	Forward: GCTTCTTCAGAGGCGACTACTGC Reverse: GCAAGCCATCTCCTCATCAGTCC	NM_009810.3	131
*caspase-9*	Forward: CCCGTGGACATTGGTTCTGG Reverse: GTGCTCGAGTTTGTCACGGT	BC056447.1	195
*AIF*	Forward: TGGGTCGAAGGCGAGTAGAG Reverse: TATGGCTTAGCGGCTCCAGT	NM_012019.3	88
*Bax*	Forward: CGTGAGCGGCTGCTTGTCTG Reverse: ATGGTGAGCGAGGCGGTGAG	LT727125.1	128
*Bcl-2*	Forward: GAGGCCTCGGAGAGGCTTTA Reverse: CGGAGGGAGTCCTTGGGAAT	NM_177410.3	95
*IL-1β*	Forward: GCTTCAGGCAGGCAGTATCACTC Reverse: GTGCTCATGTCCTCATCCTGGAAG	BC011437.1	97
*IL-18*	Forward: GGCCGACTTCACTGTACAACCG Reverse: GGTCACAGCCAGTCCTCTTACTTC	NM_008360.2	180
*TNF-α*	Forward: GCGACGTGGAACTGGCAGAAG Reverse: GCCACAAGCAGGAATGAGAAGAGG	NM_013693.3	103
*β-actin*	Forward: GCACCACACTTTCTACAATGA Reverse: ACGACCAGAGGCATACAGG	NM_007393.5	184

### Western blot analysis

Mock- and *MO*-infected MH-S cells plated on 12-well plates were collected at 24 hpi and lysed on ice with a lysis buffer (Beyotime, P0013) containing protease and phosphatase inhibitors (Beyotime, P1050) for 30 min. After centrifugation at 12,000 r/min for 5 min at 4°C, the supernatants were collected for protein quantification with a bicinchoninic acid (BCA) assay kit (Beyotime, P0012), and then mixed with appropriate amounts of SDS-PAGE loading buffer prior to boiling for 10 min. Samples were then subject to Western blot analysis as previously described [36], exploiting p53, caspase-8, caspase-12, Bax, Bcl-2, and β-actin specific primary antibodies. β-actin was regarded as a loading control. Grayscale value analyzed with ImageJ software was calculated for semi-quantisation of the target proteins.

### ROS assays

ROS levels in the mock- or *MO*-infected MH-S cells cultured in 96-well plates at 0 hpi, 6 hpi, 12 hpi, and 24 hpi were determined using a ROS detection kit (Beyotime, S0033S). The effect of NAC on ROS levels in the *MO*-infected MH-S cells at 24 hpi was also determined. In brief, 100 μL Dichloro-dihydro-fluorescein diacetate (DCFH-DA), a common probe for detecting ROS, was added to each well of 96-well plates and incubated in a 37°C incubator for 20 min. After three washes with PBS, the fluorescence intensity of each well was measured on a Synergy2 multifunctional plate reader (BioTek) at excitation wavelength (Ex)/emission wavelength (Em) = 488/525 nm.

### ELISA

MH-S cells grown in 24-well plates were pretreated with p53 inhibitor Pft-α (5 μM), caspase-8 inhibitor Z-IETD-FMK (10 μM), ROS inhibitor NAC (1:1600), or equal amount of solvent as described above, and then infected with 10 MOI of *MO*. Cell supernatants from each group at 12 hpi, 24 hpi, and 36 hpi were collected for the detection of IL-1β, IL-18, and TNF-α levels by ELISA according to manufacturer’s instructions. Standard curves were established, and the values of the samples to be tested were calculated on the basis of the generated linear regression plots.

### Cell viability assays

Cell viability of the mock- and *MO*-infected cells was detected by assays. MH-S cells seeded in 96-well plates (1 × 10^4^ cells/well) 1 day before the experiment was infected with 10 MOI of *MO*. After further incubation for 0, 6, 12, and 24 h, cells were incubated with 10% (v/v) CCK-8 in fresh medium for 1 h at 37°C. The absorbance at 450 nm was detected with a ELX808 microplate reader (BioTek instruments, Inc). Each sample was assayed in triplicate and repeated twice independently.

## Statistical analysis

The data are expressed as the mean ± standard deviation (SD) of three independent experiments and are analyzed with student’s *t* test, one-way or two-way ANOVA test. *P* values of less than 0.05 are considered to be statistically significant.

## Results

### Establishment of MO infection in MH-S cell lines

**Figure 1.**
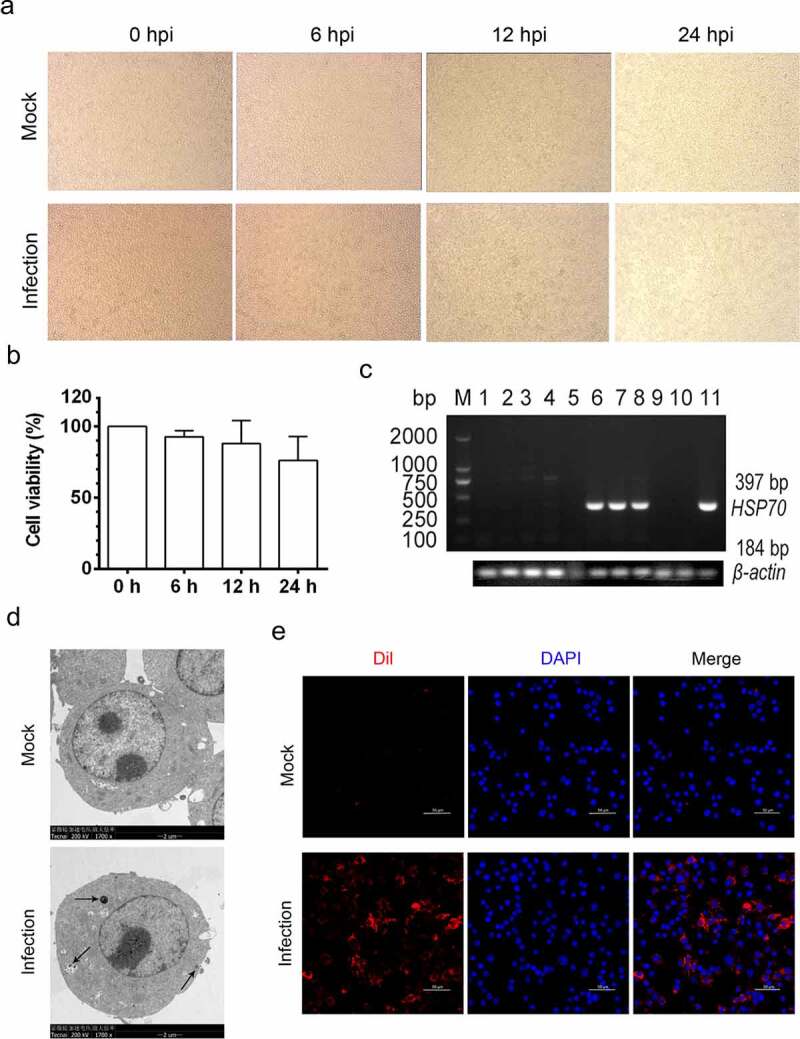
**Establishment of *MO* infection in cultured MH-S cell lines**. (a) MH-S cells cultured in 6-well plate were infected with 10 MOI of *MO* or mock infected for the indicated hours for observation of cytopathic effect. (b) Mock- and *MO*-infected cells at 0, 6, 12, and 24 hpi were collected for determination of the cell viability with CCK8 assays. (c) Mock- and *MO*-infected cells at 0, 6, 12, and 24 hpi were collected for detection of *MO*-specific *Hsp70* genes (397 bp) by reverse transcription-PCR assays. Lane 1 ~ 4 and lane 5 ~ 8: mock cells and *MO*-infected cells at 0, 6, 12, and 24 hpi, respectively; lane 9 and 10: negative control; lane 11: positive control. Mock- and *MO*-infected cells at 24 hpi were also collected for detection of *MO* infection through TEM (d) and CFM (e) analysis. For TEM (D), black arrow shows the *MO*-like structures. Bar: 2 μm. For CFM (E), *MO* was labeled with Dil probes (Orange-red fluorescence), and nucleus was stained with DAPI (blue fluorescence). Bar: 50 μm

To establish the MH-S cell model for *MO* infection *in vitro*, MH-S cells were infected with 10 MOI of *MO* for the indicated time, and then subject to detection of the status of *MO* infection. Our results show that *MO-*infected MH-S cells slightly dropped off in a time-dependent manner, with decreased adherent cells (Figure 1a). CCK8 assays also showed that the cell viability of the *MO*-infected MH-S cells decreased in a time-dependent manner despite no significant difference (Figure 1b). Transcription of the *MO*-specific *Hsp70* genes was detectable in samples from *MO-*infected MH-S cells rather than mock-infected cells at 6, 12, and 24 hpi through reverse transcription-PCR assays (Figure 1c). Moreover, *MO*-like structures were also observed in *MO-* but not mock-infected MH-S cells by TEM analysis (Figure 1d). To further verify the *MO* infection in MH-S cells, CFM analysis of the *MO-*infected MH-S cells was performed and showed that robust orange-red fluorescence indicating *MO* gathered around blue nuclei, suggesting the adherence or infection of *MO* in MH-S cells. However, orange-red fluorescence in mock-infected cells was weak (Figure 1e). Collectively, these results suggested that the MH-S cell model for *MO* infection *in vitro* has been successfully established.

### MO *induces time-dependent apoptosis in MH-S cells*

**Figure 2.**
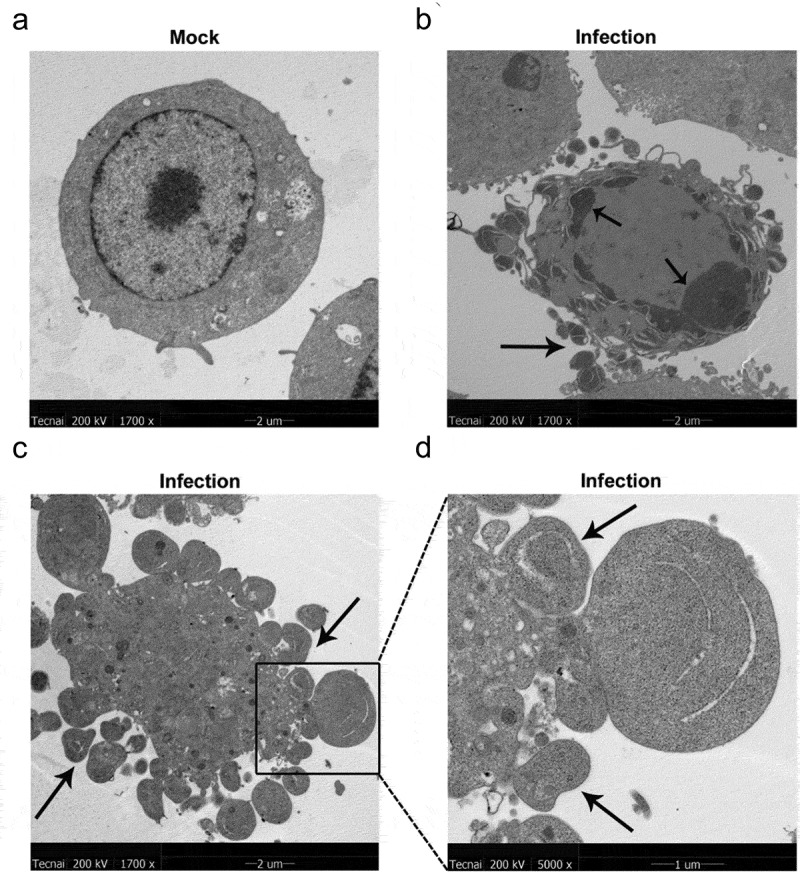
***MO* infection induces chromatin condensation and apoptotic body-like vesicles formation in MH-S cells**. MH-S cells were mock-infected (a) or infected (b-d) with 10 MOI of *MO* for 24 hours and then collected for ultrastructural observation through TEM. Short black arrow shows the perinuclear clustering of condensed chromatin. Long black arrow indicates apoptotic bodies enwrapping condensed chromatin and cytoplasmic components. Bar: 2 or 1 μm

Intriguingly, during TEM analysis, we also observed that partial MH-S cells were potentially undergoing apoptosis, as suggested by the presence of perinuclear clustering of condensed chromatin and a mass of apoptotic bodies formed by invaginated cell membranes enwrapping highly condensed chromatin and cytoplasmic components (Figure 2b-d), which are typical ultrastructures in apoptotic cells rather than in normal cells (Figure 2a). These results imply that *MO* infection induces apoptosis in the cultured MH-S cells.

**Figure 3.**
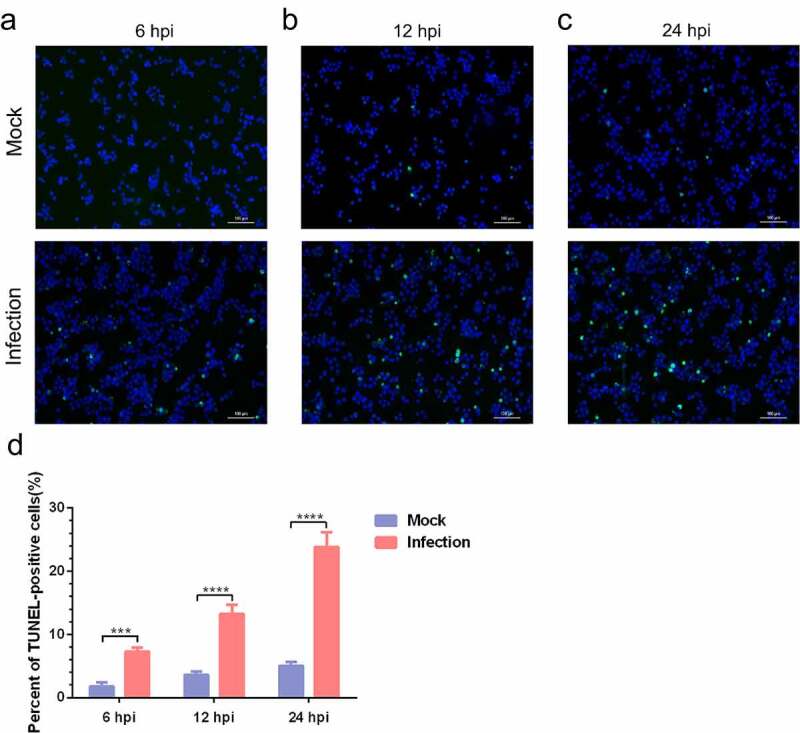
***MO* infection increases percent of TUNEL-positive cells in a time-dependent manner in MH-S cells**. (a-c) MH-S cells cultured in 6-well plates were mock-infected or infected with 10 MOI of *MO* for the indicated hours for TUNEL assays. Green fluorescence indicates the TUNEL-positive apoptotic cells, and DAPI indicates nuclei. (d) The fluorescence of cells in each group are analyzed with ImageJ software, and results are expressed as percent of TUNEL-positive cells. The data shown are expressed as mean ± SD values of three independent experiments. Two-way ANOVA tests: ***, *P* < 0.001; ****, *P* < 0.0001

**Figure 4.**
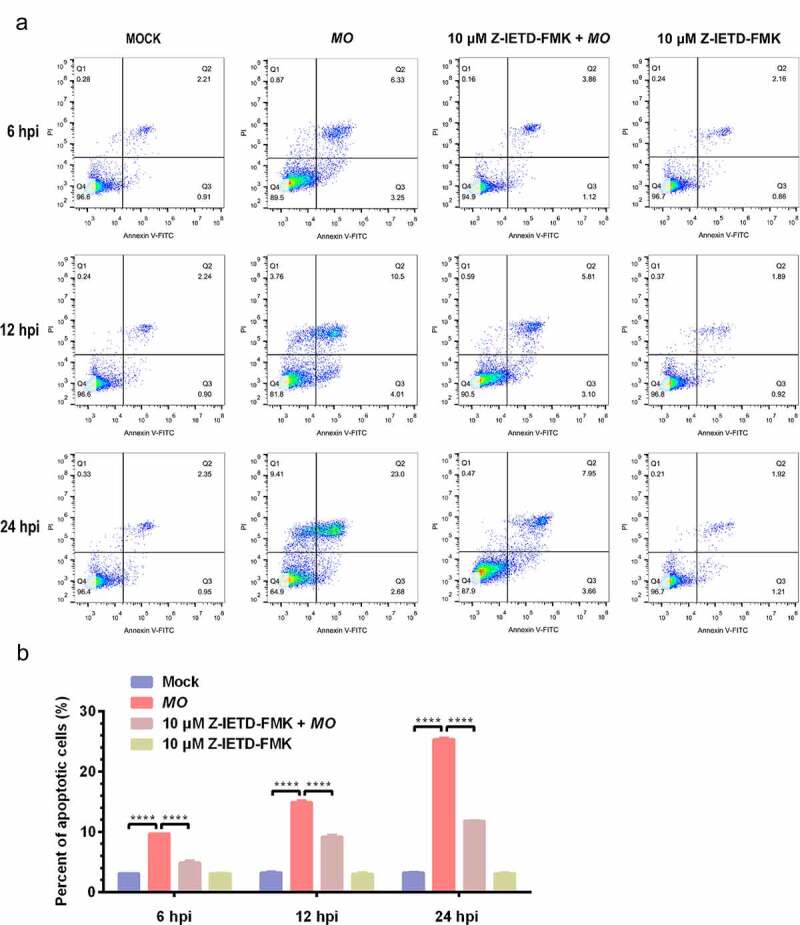
**A time-dependent increase in apoptotic rate is detectable in *MO*-infected MH-S cells**. (a-c) MH-S cells cultured in 6-well plates were mock-infected or infected with 10 MOI of *MO* in the presence or absence of 10 μM Z-IETD-FMK (a potent apoptosis inhibitor) for the indicated hours for determination of apoptosis rates by flow cytometry using an Annexin V-FITC/PI apoptosis detection kit. The percent of apoptotic cells is marked in the right side of each panel. (d) The apoptosis rates in each group were analyzed. The data shown are expressed as mean ± SD values of three independent experiments. Two-way ANOVA tests: ****, *P* < 0.0001

To further confirm *MO*-infected apoptosis in MH-S cells, time-course experiments to show the kinetics of apoptosis were conducted. *MO*-infected cells were collected at different timepoints for TUNEL and flow cytometry assays. Results of the TUNEL assays showed that the percentage of TUNEL-positive cells indicating apoptotic cells gradually increased in a time-dependent fashion. Moreover, the percentage of apoptotic cells in *MO*-infected cells was significantly larger than that in mock cells at each timepoint (6 hpi, 12 hpi, and 24 hpi) (Figure 3), suggesting a time-dependent apoptosis in *MO*-infected MH-S cells. Results of the flow cytometry assays showed that the average apoptotic rates of *MO*-infected MH-S cells at 6 hpi, 12 hpi, and 24 hpi were 9.69%, 14.90%, and 25.38%, respectively, and the average apoptotic rates of the infection group were significantly higher than those of the mock group at each timepoint (Figure 4). In addition, treatment with 10 μM Z-IETD-FMK, a potent apoptosis inhibitor, significantly decreased *MO*-induced apoptotic cells at each timepoints (Figure 4), suggesting *MO*-induced apoptosis. These results further confirm that *MO* infection induces apoptosis in MH-S cells in a time-dependent manner.

### MO activates apoptosis in MH-S cells through caspase-8-dependent extrinsic pathway and p53- and ROS-dependent intrinsic pathways

**Figure 5.**
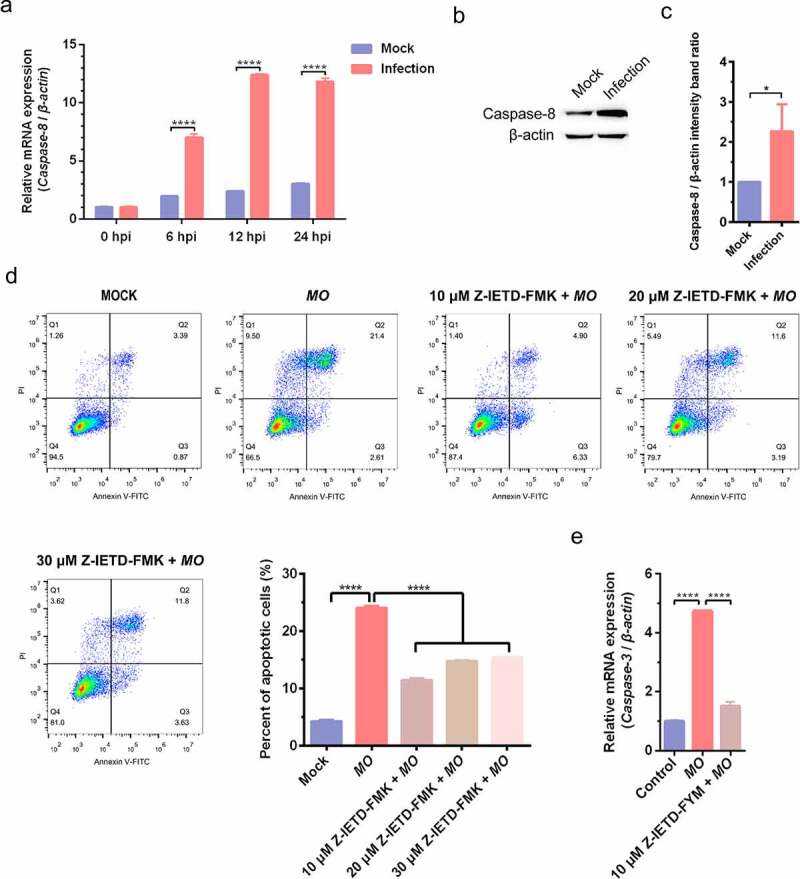
***MO* infection induces caspase-8-dependent apoptosis in MH-S cells**. (a) MH-S cells cultured in 6-well plates were mock-infected or infected with 10 MOI of *MO* for the indicated hours for relative quantification of *caspase-8* mRNA by qRT-PCR assays. Relative *caspase-8* mRNA levels normalized to the *β-actin* gene were calculated by the 2^−ΔΔCT^ method. Each sample was set up in triplicate. The data shown are expressed as mean ± SD values of three independent experiments. Two-way ANOVA tests: ****, *P* < 0.0001. (b) Samples of the mock- and *MO*-infected cells at 24 hpi were collected for detection of caspase-8 expression by Western blot as described in the “materials and methods”. β-actin is the loading control. (c) Grayscale value of the bands were analyzed with ImageJ software, and data shown are expressed as mean ± SD values of two independent experiments. Student’s *t* test: *, *P* < 0.05. (d) Cells plated in 24-well plates were pretreated with serial concentrations of caspase-8 inhibitor Z-IETD-FMK (10 μM, 20 μM, 30 μM) for 30 min, followed by infection with 10 MOI of *MO*. After 24 hours, the cells were collected for detection of apoptosis rates by flow cytometry. The apoptosis rates in each group were analyzed, and the most effective concentration of Z-IETD-FMK was selected for subsequent experiments. (e) Cells plated in 24-well plates were pretreated with 10 μM Z-IETD-FMK or equal amount of solvent prior to *MO* infection, or mock-infected for control. At 24 hpi, cells were collected for detection of *caspase-3* mRNA levels by qRT-PCR assays. Each sample was set up in triplicate. The data shown are expressed as mean ± SD values of three independent experiments. Student’s *t* test: ****, *P* < 0.0001

To further clarify the mechanisms involved in *MO*-induced apoptosis in MH-S cells, we detected the activity of the primary initiator for extrinsic apoptosis (caspase-8) and found that *MO* infection remarkably increased transcriptional levels of *caspase-8* gene in a time-dependent manner and significantly upregulated caspase-8 expression (Figure 5a-c). Transcription of *caspase-3* gene, downstream of *caspase-8*, was also significantly enhanced in *MO*-infected cells in comparison with mock-infected cells (Figure 5e). Furthermore, pharmacological inhibition of caspase-8 by 10, 20, and 30 μM Z-IETD-FMK significantly decreased *MO*-induced apoptotic rates, and the treatment with 10 μM Z-IETD-FMK also significantly suppressed *MO*-enhanced relative mRNA expression of *caspase-3* genes in MH-S cells (Figure 5d, e). These results suggest that the *MO* infection activates caspase-8-dependent extrinsic apoptosis in MH-S cells.

**Figure 6.**
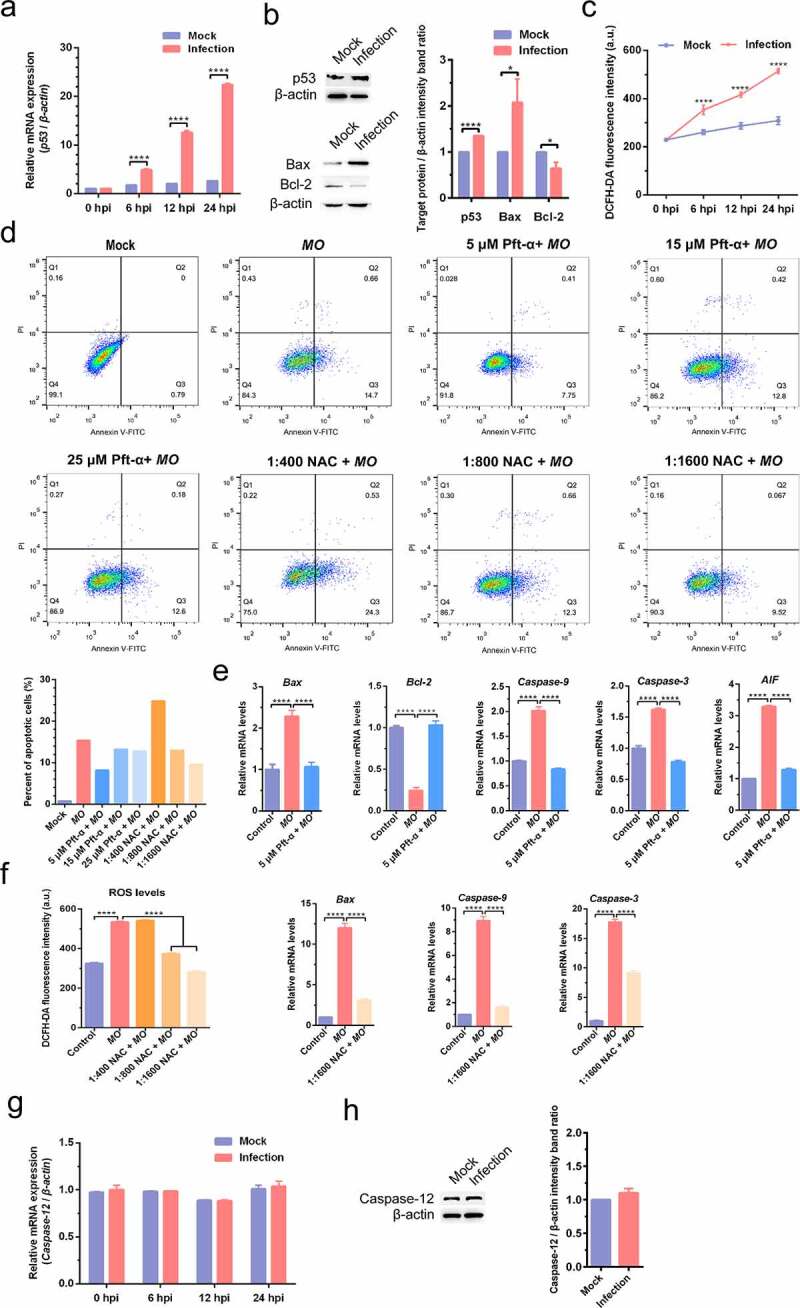
***MO* infection activates apoptosis via p53-, ROS-, but not caspase-12-dependent intrinsic pathways in MH-S cells**. (a) and (g) MH-S cells cultured in 6-well plates were mock-infected or infected with 10 MOI of *MO* for the indicated hours for relative quantification of the *p53* (A) and *caspase-12* mRNA (G) by qRT-PCR assays. Each sample was set up in triplicate. The data shown are expressed as mean ± SD values of three independent experiments. Two-way ANOVA tests: ****, *P* < 0.0001. (b) and (h) Samples of the mock- and *MO*-infected cells at 24 hpi were collected for detection of p53, Bax, Bcl-2 (B) and caspase-12 (H) expression by Western blot. β-actin is the loading control. Grayscale value of the bands were analyzed with ImageJ software, and data shown are expressed as mean ± SD values of two independent experiments. Student’s *t* test: *, *P* < 0.05, ****, *P* < 0.0001. (c) MH-S cells cultured in 6-well plates were mock-infected or infected with 10 MOI of *MO* for the indicated hours for intracellular ROS assays exploiting a DCFH-DA fluorescent probe. The fluorescence intensity of each well was measured on a multifunctional plate reader at Ex/Em = 488/525 nm. The data shown are expressed as mean ± SD values of three independent experiments. Two-way ANOVA tests: ****, *P* < 0.0001. (d) Cells plated in 24-well plates were pretreated with serial concentrations of p53 inhibitor Pft-α (5 μM, 15 μM, 25 μM) or ROS inhibitor NAC (1:400, 1:800, 1:1 600) for 30 min, followed by infection with 10 MOI of *MO*. After 24 hours, the cells were collected for detection of apoptosis rates by flow cytometry. The apoptosis rates in each group were analyzed, and the most effective concentrations of Pft-α and NAC were selected for subsequent experiments. (e) Cells plated in 24-well plates were pretreated with 5 μM Pft-α or equal amount of solvent prior to *MO* infection, or mock-infected for control. At 24 hpi, cells were collected for detection of relative mRNA expression of *Bax, Bcl-2, caspase-9, caspase-3*, and *AIF* genes by qRT-PCR assays. Each sample was set up in triplicate. The data shown are expressed as mean ± SD values of three independent experiments. One-way ANOVA tests: ****, *P* < 0.0001. (f) Cells plated in 24-well plates were pretreated with the indicated concentrations of NAC or equal amount of solvent prior to *MO* infection, or mock-infected for control. At 24 hpi, cells were collected for detection of intracellular ROS levels and relative mRNA expression of *Bax, caspase-9*, and *caspase-3* genes. The results shown are expressed as mean ± SD values of three independent experiments. One-way ANOVA tests: ****, *P* < 0.0001

We further detected effect of *MO* infection on intrinsic apoptosis pathways in MH-S cells and found that *MO* infection activated mitochondria-mediated intrinsic apoptotic pathways, as demonstrated by increased *p53* mRNA levels in a time-dependent manner (Figure 6a), upregulated p53 and Bax expression downregulated Bcl-2 expression (Figure 6b), and growing transcriptional levels of genes downstream of *p53*, including *Bax, caspase-9, caspase-3*, and *AIF*, with that accompany of declined mRNA levels of antiapoptotic *Bcl-2* (Figure 6e). Moreover, treatment with 5 μM p53 inhibitor (Pft-α) not only reduced *MO*-induced apoptotic rates (Figure 6d), but also significantly suppressed *MO*-increased transcriptional levels of *Bax, caspase-9, caspase-3*, and *AIF* genes in MH-S cells (Figure 6e). Pft-α also efficiently reversed *MO*-inhibited transcription of *Bcl-2* in MH-S cells (Figure 6e). These results suggested that p53-dependent intrinsic apoptosis occurs in response to *MO* infection of MH-S cells.

ROS also plays a crucial role in the activation of mitochondrial apoptosis. Here, we found that *MO* infection remarkably increased intracellular ROS levels in a time-dependent manner (Figure 6c) and significantly upregulated transcriptional levels of *Bax, caspase-9*, and *caspase-3* genes (figure 6f). Further study showed that treatment with ROS scavenger NAC (1:1600) not only decreased *MO*-induced apoptotic rates (Figure 6d), but also significantly inhibited *MO*-enhanced transcription of *Bax, caspase-9*, and *caspase-3* genes in MH-S cells (figure 6f). These results suggest that ROS-dependent mitochondrial apoptosis is induced in *MO*-infected MH-S cells. Taken together, our results indicate that *MO* infection induces apoptosis in MH-S cells via caspase-8-dependent extrinsic pathway and via p53- and ROS-dependent intrinsic mitochondrial pathways.

In addition, we further investigated the effect of *MO* infection on ERS-mediated intrinsic apoptotic pathways. However, no significant changes in transcriptional and translational levels of *caspase-12* gene were observed in *MO*-infected cells in comparison with mock cells (Figure 6g, h), suggesting that *MO*-induced intrinsic apoptosis is probably caspase-12-independent.

### MO induces caspase-8-, p53-, and ROS-dependent expression of proinflammatory cytokines (IL-1β, IL-18, and TNF-α) in MH-S cells

**Figure 7.**
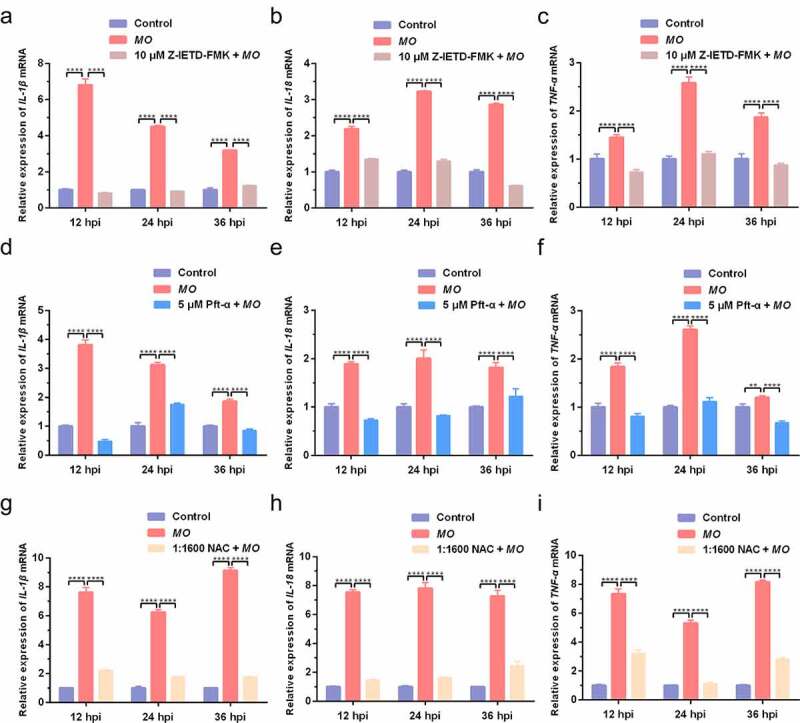
**Caspase-8-, p53-, and ROS-dependent transcriptional enhancement of proinflammatory cytokine genes in *MO*-infected MH-S cells**. MH-S cells cultured in 24-well plates were pretreated with 10 μM Z-IETD-FMK (a-c), 5 μM Pft-α (d-f), 1:1600 NAC (g-i), or equal amount of solvent followed by infection with 10 MOI of *MO*, or mock-infected for control. Cells were collected at 12 hpi, 24 hpi, and 36 hpi, respectively, and then subject to relative quantification of *IL-1β, IL-18*, and *TNF-α* mRNA by qRT-PCR assays. Relative mRNA levels normalized to the *β-actin* gene were calculated by the 2^−ΔΔCT^ method. Each sample was set up in triplicate. The data shown are expressed as mean ± SD values of three independent experiments. Two-way ANOVA tests: ****, *P* < 0.0001

**Figure 8.**
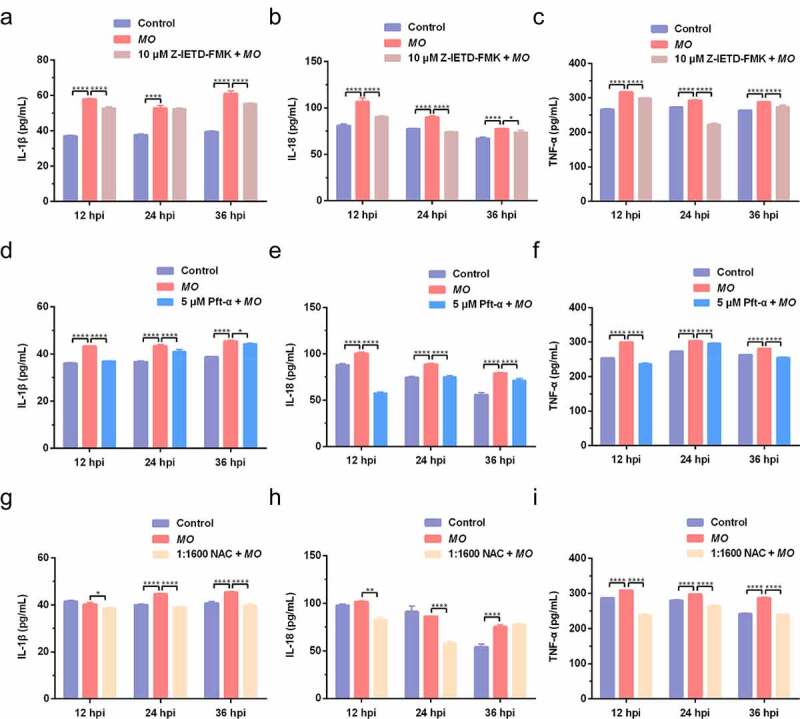
**Caspase-8-, p53-, and ROS-dependent promotion of proinflammatory cytokine secretion in *MO*-infected MH-S cells**. MH-S cells cultured in 24-well plates were pretreated with 10 μM Z-IETD-FMK (a-c), 5 μM Pft-α (d-f), 1:1600 NAC (g-i), or equal amount of solvent followed by infection with 10 MOI of *MO*, or mock-infected for control. Cell supernatants were collected at 12 hpi, 24 hpi, and 36 hpi, respectively, and then subject to determination of IL-1β, IL-18, and TNF-α secretion by ELISA assays. Standard curves were established, and values of the samples to be tested were calculated on the basis of the generated linear regression plots. The data shown are expressed as mean ± SD values of three independent experiments. Two-way ANOVA tests: *, *P* < 0.05; **, *P* < 0.01; ****, *P* < 0.0001

Apoptosis and inflammation are two important activities in *MO*-infected cells, and we further conducted time-course experiments to explore the potential relationship between *MO*-induced inflammation and apoptosis by qRT-PCR and ELISA assays. We found that *MO* infection induced significant increase in transcriptional and translational levels of proinflammatory cytokine genes (*IL-1β, IL-18*, and *TNF-α*) in MH-S cells at the indicated timepoints (12 hpi, 24 hpi, and 36 hpi) (Figures 7 and 8), except for the IL-1β secretion in Z-IETD-FMK-treated cells at 12 hpi (Figure 8g) and the IL-18 secretion in NAC-treated cells at 12 hpi and 24 hpi (Figure 8h), implying a possible function of *MO* infection in inflammatory responses. Furthermore, treatment with Z-IETD-FMK, Pft-α, and NAC remarkably downregulated *MO*-increased relative mRNA expression and secretions of IL-1β, IL-18, and TNF-α in MH-S cells at each timepoints (Figures 7 and 8), with exceptions for the IL-1β secretion in Z-IETD-FMK-treated cells at 24 hpi (Figure 8a) and the IL-18 secretion in NAC-treated cells at 36 hpi (Figure 8h). In general, our results suggested that *MO*-promoted expression of proinflammatory cytokines (IL-1β, IL-18, and TNF-α) is caspase-8-, p53-, and ROS-dependent, which is possibly linked to *MO*-induced apoptosis.

## Discussion

Generally, *MO* adheres to and colonizes airway epithelial cells and alveolar cells through the respiratory tract, where *MO* synthesizes cytotoxic products and damages cells, and even leads to apoptosis or necrosis [24,37–39]. *MO* infection not only induces epithelial cell apoptosis, but also regulates the functional activities of several immunocytes [24,25,39–41]. Nevertheless, studies on *MO*-associated apoptosis in immunocytes are rarely reported. In this study, an MH-S cell model for *MO* infection *in vitro* was first established and demonstrated by the PCR, TEM, and CFM analyses, laying foundations for our subsequent studies. Intriguingly, some likely apoptotic events, including the occurrence of condensed chromatin and apoptotic bodies were suggested by TEM analysis in the *MO*-infected MH-S cells. *MO*-induced time-dependent apoptosis was further confirmed by the TUNEL and flow cytometry assays. Our findings are similar to findings from other studies revealing that *MO* infection induces ROS-dependent apoptosis in sheep respiratory epithelial cells [24,25]. These results suggest that *MO*-induced apoptosis is a potential mechanism in the pathogenesis of *MO* infection, which remains to be verified further.

Extrinsic and intrinsic apoptosis are two well-known forms of apoptosis. Death receptor-mediated extrinsic apoptosis signaling pathway is the best-studied apoptotic pathway, which is primarily dependent on the caspase cascades initiated by caspase-8 [18,42]. Here, we found that *MO* infection-induced activation of caspase-8-dependent apoptosis in cultured MH-S cells, which is similar to the finding in a previous study showing *MO*-induced caspase-8-dependent apoptosis in sheep airway epithelial cells [24]. In our study, we found that treatment with 10, 20, and 30 μM Z-IETD-FMK inhibited *MO*-induced apoptosis, but did not show a gradient to inhibit apoptosis. The possible reason might be 20 or 30 μM Z-IETD-FMK-caused potential cytotoxicity in the context of *MO* infection, which may need further investigation. Although Z-IETD-FMK-inhibited apoptosis was not in a dose-dependent manner within our selected concentration range, Z-IETD-FMK remarkably suppressed *MO*-induced apoptosis in MH-S cells, suggesting that caspase-8 was involved in *MO*-induced apoptosis, which may not change our conclusion.

Intrinsic apoptotic pathways usually include mitochondrial and endoplasmic reticulum pathways, which are caspase-9- and caspase-12-dependent, respectively [21,43,44]. When cells undergo apoptotic stimulation such as ROS-associated DNA damage, free form of Bax in the cytoplasm and Bcl-2 homologous antagonist/killer (Bak) combine to form an oligomer complex, which is inserted into the outer mitochondrial membrane, resulting in loss of transmembrane potential and release of cytochrome c (cyt-c) into the cytoplasm, followed by activation of caspase-9 and subsequent caspase cascades [21,45]. p53-dependent activation of Bax also tilts the balance toward apoptosis [46]. Our present results revealed the p53- and ROS-driven transcriptions of *Bax, caspase-9*, and *caspase-3*, suggesting p53- and ROS-dependent intrinsic apoptosis in *MO*-infected MH-S cells. Moreover, we also observed a p53-dependent upregulation of *the AIF* mRNA levels, the first identified factor to induce caspase-independent apoptosis [47,48], implying an atypical apoptosis in a caspase-independent fashion in *MO*-infected MH-S cells. Although increasing evidence has revealed that excessive ERS activates caspase-12-dependent apoptosis [49,50] and that there exists a close relationship between ERS-mediated and mitochondrial apoptosis [51,52], obvious changes in caspase-12 expression were not detectable in our study, suggesting *MO*-induced apoptosis is caspase-12-independent. Overall, it could be seen that *MO* infection triggers multiple types of apoptosis in MH-S cells and sheep airway epithelial cells, which implies crucial roles of apoptosis in the pathogenesis of *MO* infection.

Inflammation is a conservative cellular strategy to defend the body from detrimental stimuli such as invading foreign microorganisms, and is a healing process for repairing damaged tissue as well [53,54]. It has been evidenced that several proinflammatory cytokines, including TNF-α, IL-1β, and IL-18, are capable of activating apoptosis [30,32,55]. Conversely, caspase‐8-dependent IL‐1β maturation confers a proinflammatory phenotype on apoptotic cells [56,57], and recent studies have also revealed the formation of the NOD-like receptor protein 3 (NLRP3) inflammasome via both extrinsic and intrinsic apoptosis in macrophages [58,59]. These findings suggest a complex cross-talk between inflammation and apoptosis. Here, we found that *MO* infection promotes the expression of pro-inflammatory cytokines (IL-1β, IL18, and TNF-α) in MH-S cells in caspase-8, p53-, and ROS-dependent manners, suggesting that *MO* infection may induce apoptosis by stimulating inflammatory cytokines expression; however, the underlying molecular mechanism needs further clarifications.

Increasing evidence has revealed the significant roles of IL-1β and IL-18 in pyroptosis (also known as cellular inflammatory necrosis), another type of programmed cell death mediated by gasdermin, which activates a strong inflammatory response to defend against detrimental stimuli [60,61]. A recent study revealed that apoptosis is induced in the early phases of influenza A virus infection, with a switch from apoptosis to pyroptosis in the late phases of infection in human respiratory epithelial cells [62]. The apoptosis-to-pyroptosis switch has also been implicated in the death of cancer cells [63–65]. It could be inferred from our results that *MO* infection not only induces apoptosis, but possibly triggers pyroptosis in MH-S cells suggested by remarkable upregulation of IL-1β and IL-18 secretion. Pyroptosis-associated inflammatory reactions were potentially invoked for efficient removal of *MO* and *MO*-derived metabolites, when the damaged cells were scarcely eliminated by apoptosis. Currently, our studies on the specific mechanism for *MO*-induced pyroptosis and its cross-talk with apoptosis are in progress. Answering these questions builds a bridge between *MO*-induced inflammation and apoptosis.

**Figure 9.**
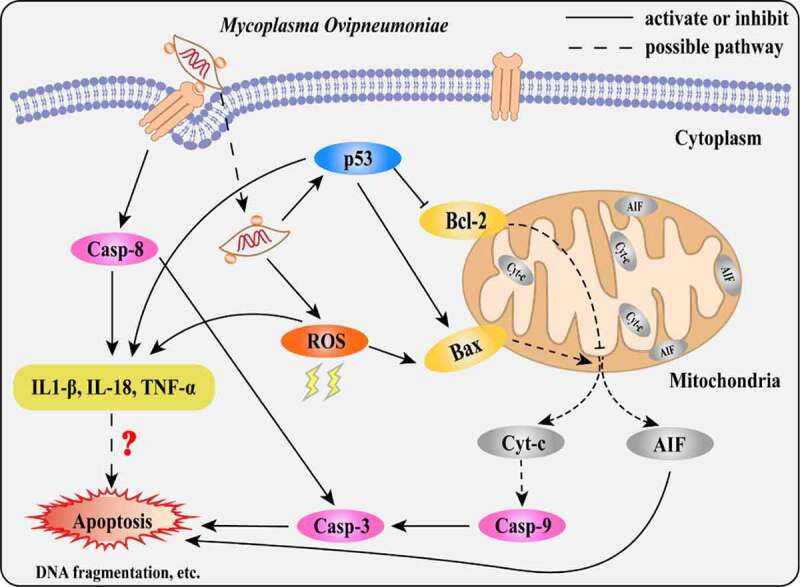
**Scheme diagram of a proposed mechanism for *MO*-induced extrinsic and intrinsic apoptosis in MH-S cells**. On the one hand, *MO* infection induces caspase-8-dependent caspase-3-mediated extrinsic apoptosis. On the other hand, *MO* infection activates apoptosis via p53- and ROS-dependent Bax/caspase-9/caspase-3-mediated intrinsic pathways, or AIF-mediated caspase-independent pathway. Furthermore, *MO* infection promotes expression of proinflammatory IL-1β, IL-18, and TNF-α in a caspase-8-, p53-, and ROS-dependent fashion, implying a potential link between *MO*-induced inflammation and apoptosis

In summary, our results suggest that *MO* infection induces caspase-8-dependent extrinsic apoptosis and p53- and ROS-dependent intrinsic apoptosis, correlating with enhanced expression of proinflammatory cytokines (Figure 9). Our findings will contribute to further clarification of the pathogenesis of *MO* infection and provide a scientific basis for the development of novel anti-*MO* strategies.

## Data Availability

The data and materials used in this study are available from the corresponding author on reasonable request.

## Abbreviations

AIF: apoptosis-inducing factor; AIV: avian influenza virus; CCU: color-changing unit; CFM: confocal fluorescence microscopy; cyt-c: cytochrome c; ELISA: enzyme-linked immunosorbent assay; ERS: endoplasmic reticulum stress; FBS: fetal bovine serum; hpi: hours post-infection; Hsp70: heat shock protein; IFN-γ: interferon-γ; IL-1β/18: interleukin-1β/18; MAPK: mitogen-activated protein kinases; *MO: Mycoplasma ovipneumoniae*; NLRP3: NOD-like receptor protein 3; PCD: programmed cell death; p53: tumor protein 53; SARS-CoV-2: severe acute respiratory syndrome coronavirus-2; SD: standard deviation; TEM: transmission electron microscopy; TNF-α: tumor necrosis factor-α; TNFR: tumor necrosis factor receptor; TUNEL: terminal deoxynucleotidyl transferase-mediated dUTP-biotin nick end labeling; ROS: reactive oxygen species; UPR: unfolded protein response.
